# Urinary Biomonitoring of Mycotoxins in Spanish Adults: Predictors of Exposure and Health Risk Evaluation

**DOI:** 10.3390/toxics13100856

**Published:** 2025-10-10

**Authors:** Borja Peris-Camarasa, Clara Coscollà, Pablo Dualde, Olga Pardo

**Affiliations:** 1 Foundation for the Promotion of Health and Biomedical Research in the Valencian Region, FISABIO-Public Health, Avda. Cataluña, 21, 46020 Valencia, Spain; borja.peris@fisabio.es (B.P.-C.); pablo.dualde@fisabio.es (P.D.); 2 Department of Analytical Chemistry, University of Valencia, Doctor Moliner, 50, 46100 Burjassot, Spain; olga.pardo@uv.es

**Keywords:** mycotoxins, risk assessment, human biomonitoring, urine, Spanish adults

## Abstract

Mycotoxins are toxic secondary metabolites produced by fungi, frequently present in food and representing significant health hazards. Exposure occurs through the consumption of contaminated foods or animal-derived products from livestock fed with contaminated feed. This study evaluated internal exposure to twelve mycotoxins in 492 first-morning urine samples from adults, aged 18–65 years, in the Valencian Community, Spain. Samples were analysed using a “dilute-and-shoot” approach followed by UHPLC-MS/MS. Aflatoxins (AFs) were the most frequently detected, with a geometric mean (GM) of 1.17 ng/mL and a 95th percentile (P95) of 6.04 ng/mL. Alternariol (AOH), present in 63% of samples, showed high concentrations (GM: 0.98 ng/mL; P95: 4.74 ng/mL). Emerging mycotoxins such as alternariol monomethyl ether (AME), citrinin (CIT), and sterigmatocystin (STER) were also considered due to their potential health impacts. Exposure levels correlated with variables including sex, age, annual income, smoking status, and recent consumption of meat and cereals. Probable daily intakes (PDIs) were estimated from urinary concentrations to support risk assessment. Hazard Quotients (HQs), Margins of Exposure (MOEs), the Hazard Index (HI) and the total Margin of Exposure (MOET) were calculated to evaluate the risk associated with mycotoxin exposure. Findings suggest that potential health risks cannot be excluded.

## 1. Introduction

Globalization, industrial food production, and climate change have contributed to increased exposure to emerging contaminants, posing significant risks to human health [1]. Among these, mycotoxins are of particular concern within the European Union (EU), as evidenced by notifications from the Rapid Alert System for Food and Feed (RASFF) between 2021 and 2023 [2]. During this period, mycotoxins ranked as the third most notified hazard category [2]. Produced as secondary metabolites by fungi, these naturally occurring toxins are also considered among the most critical contaminants due to their potential chronic toxicity [3,4].

Mycotoxin contamination can occur at various stages, as fungal growth is possible during harvest, storage, and even in ready-to-eat food [5]. Environmental factors such as high temperatures and humidity play a crucial role in fungal proliferation, making this issue especially relevant in Mediterranean countries such as Spain [6,7]. Mycotoxin exposure occurs either directly through the consumption of contaminated agricultural products (cereals, fruits, vegetables, etc.) or indirectly through animal-derived foods (egg, milk, etc.) and fish that have ingested contaminated feed [4,8,9,10,11]. Due to their chemical stability, most mycotoxins persist even after food processing [12,13], making them a significant concern for both food safety and the economy [14]. Additionally, conventional treatments, such as washing, are ineffective in eliminating these toxins [15].

Exposure to these toxins has been linked to various toxicological effects, including carcinogenic and mutagenic properties, kidney and liver damage, neurotoxicity and immunosuppression. Furthermore, they can induce estrogenic, cytotoxic, and inflammatory reactions, as well as disorders affecting the gastrointestinal, renal, urogenital, and nervous systems [3,15,16,17,18,19,20].

Aflatoxins (AFs), zearalenone (ZEN) and ochratoxin A (OTA) are among the most prevalent and toxic mycotoxins. AFs are toxic compounds produced by fungi of the genus *Aspergillus*, with the most common types being aflatoxins B_1_ (AFB_1_), B_2_ (AFB_2_), G_1_ (AFG_1_), G_2_ (AFG_2_), and M_1_ (AFM_1_). AFB_1_ is the most potent natural carcinogen and has been categorised as “carcinogenic to humans” (group 1) by the International Agency for Research on Cancer (IARC) [21]. ZEN, produced by *Fusarium* species, exhibits estrogenic activity that can negatively impact the genital organs and reproductive system [18,22]. Due to these effects, the European Food Safety Authority (EFSA) assigned relative potency factors to ZEN and its metabolites and established a tolerable daily intake (TDI) of 0.25 µg/kg-bw/day [23]. OTA is produced by fungi from the *Aspergillus* spp. and *Penicillium* spp. and is associated with nephrotoxicity and carcinogenicity [24,25]. Given evidence from animal studies, IARC has categorised OTA as a “potential human carcinogen” (group 2B), as conclusive human evidence is lacking [26]. Additionally, EFSA has defined a benchmark dose lower confidence limit for a 10% response (BMDL_10_) of 14.5 µg/kg-bw/day [27]. These mycotoxins are strictly regulated under European legislation (EC 915/2023), which establishes allowable levels in food products [20], as diet is recognised as the primary route of mycotoxins exposure [6,7]. Due to these stringent regulations and the implementation of hazard analysis and critical control point (HACCP) strategies within the food industry, legislated mycotoxins are generally not expected to be present in high concentrations in food products [28].

Beyond the major mycotoxin classes, certain emerging mycotoxins including *Alternaria* toxins (AOH and AME), citrinin (CIT) and sterigmatocystin (STER) are gaining attention, as their presence in food [5]. These lesser-known compounds are not routinely monitored or regulated in foods and other matrices, and data on their toxicological profiles remain limited [29,30]. In vitro studies suggest that some emerging mycotoxins can disrupt intracellular ion homeostasis or induce genotoxic, mutagenic and enzyme-modulating effects [29,31]. In view of the potential for toxicity, EFSA has applied the threshold of toxicological concern (TTC) approach to assess dietary exposure risks, determining safe intake levels of 2.5 ng/kg-bw/day for AME and AOH [32].

Traditional mycotoxin exposure assessment relies on the analysis of food products combined with estimates of average dietary intake. While these measures are essential for ensuring food safety, they come with limitations, such as challenges in collecting accurate dietary intake data, the heterogeneous distribution of mycotoxins in foods, and the inability to reflect individual exposure levels. Additionally, factors such as bioavailability, toxicokinetics, and the difficulty of sampling food from subsistence farming communities further complicate exposure assessment [33,34,35]. In contrast, human biomonitoring (HBM) offers a valuable alternative for evaluating mycotoxin exposure by addressing these constraints. Analysing mycotoxins and/or their metabolites in human urine provides a more precise assessment of individual exposure, as it accounts for variations in absorption, distribution, metabolism, and excretion [36,37], under the assumption the uncertainty and individual heterogeneity of all steps are not too large. Furthermore, HBM enables the identification of targeted corrective measures within specific geographical or social contexts [38].

Mycotoxins encompass a diverse range of compounds with distinct biochemical properties that affect their metabolic pathways. Urine has been identified as a primary excretion route for mycotoxins, making it the preferred biological matrix for detecting these toxins and their metabolites due to its ease of collection and non-invasive nature. Among AFs, AFM_1_ is the most commonly analysed metabolite, formed via hydroxylation of AFB_1_ at C-7 in mammals [25,26,39]. After exposure, ZEN undergoes rapid absorption and is mainly metabolised through reduction reactions, producing metabolites such as zearalanone (ZAN), α-zearalenol (α-ZEL), β-zearalenol (β-ZEL), α-zearalanol (α-ZAL) and β-zearalanol (β-ZAL) [11]. OTA is a toxic compound characterized by fast absorption and slow elimination [11]. It is excreted through both urine and feces after biliary excretion [40]. The primary metabolic pathways of OTA include hydrolysis, hydroxylation, lactone opening, and conjugation. The main metabolite, ochatoxin alpha (OTα), is produced via cleavage of the peptidic bond [11]. This OTA metabolite and ochratoxin B (OTB), are considered less toxic than the parent compound [41]. However, while hydroxylated and glucuronide derivatives of OTA have been suggested as potential metabolites, few studies [5,11] have reported their occurrence in urine. Despite their external control, the pharmacokinetics of both ZEN and OTA remain insufficiently characterized, emphasizing the need for further validation of urinary biomarkers for these mycotoxins [19]. Similarly, although some studies have investigated emerging toxins such as AOH, AME and STER, their metabolic pathways are still not well understood. Finally, for CIT, dihydro-citrinone has been identified as a urinary metabolite [42,43], reinforcing its potential as a biomarker, alongside CIT itself, for biomonitoring applications.

HBM has been widely applied in studies worldwide to assess urinary biomarkers of mycotoxin exposure in adult populations [29,37,44,45,46,47,48,49,50]. Many of these studies analysed only a limited spectrum of mycotoxins in urine samples [11,19,22,31,51,52,53], highlighting the need for more comprehensive research. In Spain, although several HBM studies have examined mycotoxin exposure, they often involved small sample sizes (20–56 participants) [11,18,54] or focused on specific subpopulation groups, such as women [5], rather than a representative sample of the adult population. Therefore, the main objective of this study is to examine urinary mycotoxin levels in a representative sample of the Spanish adult population (aged 18–65 years), particularly in residents of the Valencian Community (Spain). The aim is to provide insight into current mycotoxin exposure levels and improve our understanding of the associated risks. Furthermore, this study also evaluates the risks linked to mycotoxin occurrence and explores potential predictors of exposure. The obtained data will be useful in establishing reference values and identifying potential mycotoxin exposure routes.

## 2. Materials and Methods

### 2.1. Study Design

This study is part of the cross-sectional HBM project BioMoVal. Detailed descriptions of its objectives, participant recruitment, data and sample collection methods, and other methodological details have been previously published by Peris-Camarasa et al. [55]. Briefly, in 2021, 527 adults aged 18–65 years from the Valencian Community (Spain) participated by providing a first-morning spot urine sample and completing a structured questionnaire collecting socio-demographic information, dietary habits, and other relevant lifestyle factors.

### 2.2. Ethical and Confidentiality

The BioMoVal project was approved by the Ethics Committee for Research of the General Directorate of Public Health and Centre for Advanced Research in Public Health of the Valencian Government. Prior to the collection of data and biological samples, all participants provided written informed consent. Personal information was managed with strict confidentiality and handled securely throughout processing and transfer. All data management procedures adhered to applicable national legislation and European Union (EU) ethical and legal frameworks, including compliance with the General Data Protection Regulation.

### 2.3. Determination of Mycotoxins in Urine

In total, twelve different mycotoxins were determined in human urine samples, encompassing a broad range of toxicological classes. These included five aflatoxins (B_1_, B_2_, G_1_, G_2_ and M_1_), OTA, ZEN and ZAN, AOH and AME, CIT and STER purchased from Sigma-Aldrich (Barcelona, Spain). The detection and quantification of these compounds were conducted using the analytical method previously published by Peris-Camarasa et al. [56].

In summary, after thawing at room temperature and centrifuging the urine samples, 800 µL of the resulting supernatant from each sample was dispensed into a 96-well plate and spiked with 50 µL of the IS working solution. Then, the volume was adjusted to 1 mL with water:acetonitrile (70:30 *v*/*v*) supplied by VWR Prolabo (Barcelona, Spain). Finally, 10 µL was injected into a Vanquish UHPLC system from Thermo Fisher Scientific Inc. (San Jose, CA, USA), equipped with an ACQUITY UPLC HSS T3 C_18_ column (100 mm × 2.1 mm, 1.8 µm, 100Å) from Waters^®^ (Milford, MA, USA) and coupled to a TSQ Altis^TM^ Triple Quadrupole Mass Spectrometer from Thermo Fisher Scientific^®^ Inc. Mass analysis was performed using a heated electrospray ionization source (H-ESI) in positive and negative ion modes and selected reaction monitoring (SRM) mode. The procedure and criteria for quality assurance/quality control (QA/QC) and method validation are fully described Peris-Camarasa et al. [56]. The spectrometric parameters, including precursor/product ion pairs, collision energies and others necessary for identifying different target compounds were also obtained (see Supplementary Table S1). Additionally, a chromatogram of each target compound was included to demonstrate their accurate quantification in urine samples using this methodology (see Supplementary Figure S1).

### 2.4. Statistical Analysis

Data analysis was performed using IBM SPSS Statistics version 22.0 and R software version 4.5. The maximum likelihood estimation technique (MLE), as recommended by EFSA [57], was applied to estimate the concentration of unquantified compounds (<LoQ). Descriptive statistics were calculated, including detection frequency (DF, %), arithmetic and geometric means (AM and GM), 25th, 50th, 75th and 95th percentiles (P25, P50, P75, and P95), as well as minimum, maximum, and standard deviation (SD). As reported in previous HBM [58,59,60], SG-adjusted and Cre-adjusted levels were calculated to correct urinary dilution effects. Additionally, demographic and lifestyle information from participant questionnaires was analysed to characterise the study sample.

Spearman’s correlation values were calculated to assess bivariate associations between individual urinary mycotoxin levels, following the coefficient interpretation criteria proposed by Akoglu [61]. This represents the first application of this test for assessing inter-mycotoxin correlations, as prior studies primarily used it to examine associations with exposure variables or to compare urine sample types [46]. Subsequently, in accordance with HBM4EU recommendations [62], multiple linear regression models were built to evaluate the influence of exposure variables on urinary mycotoxin concentrations. Based on structural and toxicological similarities, certain mycotoxins were grouped into families: AFs were analysed as their sum (∑AFs), and the urinary levels of ZEN and its metabolite ZAN were combined as ∑ZENs. To ensure statistical robustness, models were created for both grouped mycotoxin families and individual compounds with detection rates surpassing 25%, as a consensus-based inclusion criterion.

To address the non-normal distribution of raw concentration data (ng/mL), a logarithmic transformation was applied to the dependent variable. Model selection was performed using a forward stepwise approach guided by the corrected Akaike information criterion (AICc) [63], and the statistical significance was set at 0.05. Additionally, in alignment with the approach of Peris-Camarasa et al. [55,60], SG was included as an independent variable in the regression models to account for hydration-related variation in urinary concentrations, as recommended by Barr et al. [64]. Following model construction, diagnostic tests were conducted to ensure that regression assumptions were satisfied, including checks for normality, independence of residuals, homoscedasticity, and multicollinearity. These assessments involved the Kolmogorov–Smirnov test, Durbin-Watson statistic, scatter plot evaluations, and analysis of tolerance and condition indices. Finally, although three approaches were used to report urinary levels (unadjusted, Cre-adjusted and SG-adjusted), emphasis was placed primarily on SG-adjusted results, particularly for the goal of risk assessment.

### 2.5. Exposure and Risk Assessment

In this study, mycotoxin exposure was assessed using an internal dose approach, as traditional assessment methods do not fully capture exposure characteristics at the individual level [46]. To evaluate the potential risk associated with the observed exposure level, the PDI was calculated under the assumption that dietary intake of contaminated food was the primary source of human exposure. It is important to note that exposure and risk assessments were performed only for compounds with detection frequencies exceeding 25%, specifically OTA, AOH, ∑AFs, and ∑ZENs.

For each mycotoxin or mycotoxin family, reverse dosimetry was applied, given the absence of BEs or German HBM values (HBM—I and HBM—II) for these compounds. Urinary mycotoxin concentrations were converted into intake estimates, expressed as µg/kg-bw/day. A deterministic intake mass balance approach was employed, incorporating mycotoxin concentrations (C) in urine, both unadjusted (ng/mL) and adjusted using SG values (ng/mL) and urinary Cre levels (µg/g Cre), at both GM and P95 distribution levels. The intake calculation also considered the estimated total urinary volume excreted over 24 h (V_24h_), the total daily urinary Cre excretion (Cre_24h_), as well as the body weight (kg) and the urinary excretion rate (F_UE_, %), as follows:

(1) $$PDI\left(µg/kg-bw/day\right)=\frac{C\cdot V_{24h}\cdot 100}{F_{UE}\cdot BW}$$

(2) $$PDI\left(µg/kg-bw/day\right)=\frac{C\cdot {Cre}_{24h}\cdot 100}{F_{UE}\cdot BW}$$

All data regarding the participants (body weight and urinary concentrations) were considered at the individual level. However, the daily urinary volume (V_24h_) was taken as the population average (1.65 L/day) due to the equal sex distribution between males (1.7 L/day) and females (1.6 L/day) [65]. Similarly, daily urinary Cre excretion (Cre_24h_) was normalised by the mean body weight (kg), age (years old) and height (cm) of the study population (1.48 g Cre/day), calculated as shown by Mage et al. [66]. Finally, the F_UE_ values were taken from Jager et al. [67] for AFs (1.3%), Warth et al. [68] for ZEN and metabolites (9.4%), Studer-Rohr et al. [69] for OTA (2.5%), and Puntscher et al. [70] for AOH (8.3%).

The EFSA established a TDI of 0.25 µg/kg-bw/day for ZEN and its metabolites [23]. In the case of AOH and AME, EFSA has not established a formal TDI; however, a TTC of 2.5 ng/kg-bw/day has been proposed for these compounds due to insufficient toxicological data [32].

To evaluate the potential health risks associated with mycotoxin exposure, the PDIs, calculated at both GM and P95 distribution levels, were compared with the corresponding existing safe intake thresholds (HBGV) by calculating their respective HQs (Equation (3)), since there are no HBM reference values established for the mycotoxins in biological samples [46]. This comparison allowed for a preliminary assessment of whether the estimated exposures may pose a health concern.

(3) $$HQ\left[dimensionless\right]=\frac{PDI\left(µg/kg-bw/day\right)}{HBGV\left(µg/kg-bw/day\right)}$$

When the HQ was lower than 1, the exposure was considered to be within safe limits [71].

On the other hand, in cases where HBGV values are not available in the literature, the EFSA recommends the use of the MOE approach to estimate the potential health risk of a substance [72]. This approach is also recommended to perform risk characterization of potential carcinogenic substances [71]. The MOE is defined as the ratio between the BMDL_10_ and the PDI (Equation (4)). For the present analysis, BMDL_10_ values of 0.40 µg/kg-bw/day for AFs and 14.5 µg/kg-bw/day for OTA were applied, based on EFSA guidance [27,73].

(4) $$MOE\left[dimensionless\right]=\frac{{BMDL}_{10}\left(µg/kg-bw/day\right)}{PDI\left(µg/kg-bw/day\right)}$$

An MOE greater than 10,000 indicates that the substance poses a negligible risk to public health [72].

Simultaneous exposure to multiple chemical hazards can result in toxic effects even at low concentrations, due to potential synergistic interactions [74,75]. This presents a significant challenge for conventional risk assessment approaches, which often evaluate substances in isolation. Given that consumers are frequently exposed to multiple mycotoxins through various dietary and environmental routes [68], there is increasing concern regarding the cumulative health risks posed by such co-exposures.

Research indicates that even when individual toxins are present at concentrations below established safety thresholds, their combined exposure may still lead to adverse effects [76,77]. Despite this, traditional biomonitoring strategies have largely focused on single mycotoxins, thereby overlooking the potential risks associated with mixture toxicity. To address this gap, several models have been developed to better characterise cumulative risk, including the HI and MOET [78,79,80]. The HI model is typically used for mixtures containing substances that are not genotoxic or carcinogenic and involves summing the individual HQs for each compound in the mixture (Equation (5)). In contrast, the MOET approach (Equation (6)) is recommended for evaluating genotoxic and carcinogenic compounds and is widely applied in cumulative risk assessment frameworks [71].

(5) $$HI=\sum {HQ}_{i}$$

(6) $$MOET=\frac{1}{\sum \frac{1}{{MOE}_{i}}}$$

Similarly to the interpretation of HQs, a HI value greater than 1 may indicate a potential health concern. For MOET, a value exceeding 100 is generally considered to represent an adequate margin of safety for human health [81,82].

## 3. Results

### 3.1. Population Characteristics

Urinary concentrations of twelve different mycotoxins were analysed in samples collected from a representative Spanish adult population. After applying exclusion criterion based on urinary creatinine (Cre) levels, a total of 492 urine samples were included in the final analysis. Specifically, 35 participants with Cre concentrations outside the recommended range of 30–300 mg/dL were excluded, in accordance with the criterion established by Barr et al. [64].

Participant characteristics, including socio-demographic variables, lifestyle factors, and dietary habits, are presented in Table 1. The median age of participants was 43 years, with distributions by sex, age group, and geographic location broadly reflecting the demographic composition of the target population. In terms of economic status, the majority of individuals reported a moderate annual per capita income (20,000–59,999 €, 53.8%). Regarding, lifestyle behaviours, most participants identified as non-smokers (60.2%). Finally, in terms of urinary parameters, the average specific gravity (SG) was 1.020, with values ranging from 1.002 to 1.034. Mean urinary Cre level was 127, with a standard deviation (SD) of 60 mg/dL, indicating a generally acceptable range for biomonitoring purposes.

### 3.2. Occurrence and Co-Occurrence of Mycotoxins in Urine

Descriptive statistics for urinary mycotoxin concentrations are summarised in Table 2, with results presented using raw data and SG-adjusted concentrations. Cre-adjusted concentrations are also obtained (see Supplementary Table S2). Among the various mycotoxins evaluated, AFs emerged as the most prevalent mycotoxin family in these urine samples. Specifically, the biomarkers AFB_1_, AFB_2_, AFG_2_ and AFM_1_ exhibited DFs exceeding 75%, highlighting their widespread occurrence in the study population. In contrast, AFG_1_ was detectable in only a minority of samples, with a detection frequency (DF) of just 10.8%.

Apart from AFs, other notable mycotoxins included ZAN and AOH, which were identified in 57.1% and 63.0% of the samples, respectively. Among these, AOH was detected at the highest SG-adjusted urinary concentrations, with a geometric mean (GM) of 0.98 ng/mL and a 95th percentile (P95) of 4.74 ng/mL. This was followed by AFG_2_ (GM: 0.36 ng/mL, P95: 4.45 ng/mL) and AFM_1_ (GM: 0.22 ng/mL, P95: 1.65 ng/mL). For mycotoxins such as AFG_1_, AME, CIT, and STER, which exhibited low DFs below 20%, certain statistical parameters were not calculated due to insufficient data. The cumulative concentrations of specific mycotoxin groups were also assessed. The total sum of aflatoxins (∑AFs) ranged from 0.03 to 21.4 ng/mL, while the combined levels of zearalenone-related biomarkers (∑ZENs) varied from below the limit of quantification (<LoQ) to 55.5 ng/mL.

Importantly, co-exposure to multiple mycotoxins was commonly observed among participants. Nearly half (46.1%) of the urine samples contained more than five different mycotoxins, and 6.5% (N = 32) exhibited simultaneous detection of at least seven distinct compounds. Notably, 28 urine samples contained detectable concentrations of all targeted AFs, and 242 samples tested positive for four out of the five main AFs (AFB_1_, AFB_2_, AFG_2_, and AFM_1_). Regarding the predominant individual mycotoxins, AFB_1_, ZEN, and OTA, a total of 37 individuals (7.5%) demonstrated concurrent exposure to all three, indicating a potentially heightened health risk from cumulative exposure.

### 3.3. Statistical Relationships and Influence of Exposure Variables

Spearman’s correlation matrices for urinary mycotoxin concentrations, including unadjusted, SG-adjusted and Cre-adjusted values, were computed (see Supplementary Tables S3–S5). Statistically significant correlations were observed among various AFs, with coefficients ranging from 0.128 to 0.506 in unadjusted data, 0.109 to 0.476 for SG-adjusted values, and 0.105 to 0.484 for Cre-adjusted results. These correlations were generally classified as weak to moderate in strength, according to the criteria detailed by Akoglu [61]. Notably, the correlation between AFG_1_ and AFG_2_ was not statistically significant across any adjustment method. Additionally, among AFs, AFB_1_, AFB_2_ and AFG_1_ also displayed significant positive associations with other mycotoxins, including ZEN (0.094–0.202), OTA (0.117–0.229), CIT (0.123–0.245) and STER (0.215–0.297).

Moreover, a significant direct correlation was found between ZEN and OTA (0.124–0.193, *p* < 0.01), suggesting potential co-exposure patterns. Conversely, no significant correlations were observed between ZAN and its parent compound ZEN, possibly due to differences in DFs and urinary levels, suggesting a high conversion rate of ZEN to ZAN. Among emerging mycotoxins, AOH exhibited significant but weak correlations with AFB_1_ (0.261–0.311) and ZEN (0.095–0.109). Furthermore, CIT and STER were significantly correlated (0.202–0.207), indicating potential concurrent exposure. In contrast, no significant correlations were found between Alternaria toxins like AOH and AME. All these variations in mycotoxin associations may be attributed to their widespread occurrence.

Table 3 presents the results of the multiple linear regression models conducted for each individual mycotoxin and mycotoxin family. Overall, these models demonstrated limited explanatory power, accounting for between 3.3% and 17.0% of the variance (R^2^) in urinary mycotoxin concentrations. As anticipated, all urinary mycotoxin levels were significantly influenced by SG values (β ranging from 6.907 to 25.583), highlighting the critical role of urine dilution as a physiological confounder in biomarker analyses.

Participant characteristics also contributed to the variability observed. Notably, sex and annual per capita income were associated with urinary concentrations of ΣAFs, while age was a determinant of urinary ΣZENs and OTA levels. Specifically, females exhibited lower ΣAFs concentrations in their urines compared to males (β = −0.160). Furthermore, participants from lower-income households tended to present higher ΣAFs levels, with standardized β coefficients of 0.156 for moderated incomes (20,000–59,999 €) and 0.226 for low incomes (<19,999 €). Although weak, age had a direct positive effect on ΣZENs (β = 0.009) and OTA (β = 0.003) concentrations.

Smoking status emerged as a significant predictor of both ΣAFs (β = 0.109) and OTA (β = 0.155, *p* < 0.001) levels, with active smokers showing substantially elevated urinary concentrations relative to non-smokers and passive smokers. In terms of dietary habits, recent consumption (within 24 h) of meat, particularly processed meat and chicken, was positively associated with urinary ΣAFs concentrations (β = 0.657 and 0.381, respectively). Additionally, cereal intake was positively related to urinary levels of ΣZENs (β = 5.479) and AOH (β = 1.888).

### 3.4. Risk Assessment

Dietary exposure assessments were performed by estimating probable daily intake (PDI) values exclusively for the most prevalent mycotoxins detected in the target population: ΣAFs, ΣZENs, OTA and AOH. PDI values were derived for the whole studied population according to their urinary biomarker concentrations, applying Formulas (1) and (2) (Section 2.5) across raw, SG-adjusted and Cre-adjusted datasets. Table 4 presents the PDIs at both GM and P95 distribution levels, representing average and high-end exposures, respectively.

In accordance with the urinary concentration data, ΣAFs exhibited the highest PDI values among the assessed mycotoxins, ranging from 1.27 to 2.03 µg/kg-bw/day at GM, and from 6.26 to 9.77 µg/kg-bw/day at P95. Although AFB_1_ is classified as “carcinogenic to humans” by IARC [21], no biomonitoring equivalents (BEs) or established health-based guidance values (HBGVs) are currently available. As a result, health risk was assessed using the margin of exposure (MOE) approach, as described in Formula (4) (Section 2.5), in line with the EFSA’s guidance [72]. Given its toxicological profile, exposure should be reduced to as low as reasonably achievable [83]. MOE values for AFs were extremely below the safety threshold of 10,000, and nearly all participants (N = 455, 92.5%) exhibited PDI values exceeding the benchmark dose lower confidence limit associated with a 10% response of an adverse effect (BMDL_10_) value (0.4 µg/kg-bw/day), highlighting a potential risk to human health.

PDI values for ΣZENs ranged from 0.01 to 0.02 µg/kg-bw/day at GM, and from 0.36 to 0.65 µg/kg-bw/day at P95. While mean hazard quotients (HQs) were below 1, HQs at P95 exceeded this threshold, suggesting that a substantial portion of the target population may be at risk. Specifically, 81 participants (16.5%) had PDI values for ΣZENs above the established TDI (0.25 µg/kg-bw/day).

Regarding OTA, exposure estimated ranged from 0.02 to 0.04 µg/kg-bw/day at GM and from 0.11 to 0.16 µg/kg-bw/day at P95. In this case, MOE values fell below the critical threshold of 10,000.

For AOH, PDI values ranged from 0.17 to 0.27 µg/kg-bw/day at GM, and from 0.85 to 1.39 µg/kg-bw/day at P95. In the absence of established HBGVs for Alternaria toxins and limited toxicological data, exposure was evaluated against the TTC value (2.5 ng/kg-bw/day) established by the EFSA. None of the participants had an exposure level below this TTC value, with mean exposure levels exceeding the TTC by 67- to 107-fold, underscoring a substantial toxicological concern.

Finally, cumulative risk assessment was performed by calculating hazard index (HI) and total margin of exposure (MOET) values to evaluate the potential effects of simultaneous exposure to multiple mycotoxins. Although these mycotoxins may act through distinct toxicological mechanisms, possible synergistic effects should also be considered. According to their respective individual risk assessment indicators, HI was applied for ZENs and AOH, while MOET was used for AFs and OTA. In line with the individual risk assessment results, both HIs and MOETs exceeded or fell below their respective thresholds, indicating potential concerns regarding multi-mycotoxin exposure.

## 4. Discussion

### 4.1. Occurrence and Co-Occurrence of Mycotoxins in Urine

The present study examined urinary concentrations of twelve mycotoxins in a representative sample of the Spanish adult population to assess exposure risks. While capturing long-term exposure ideally requires multiple samples over time, this is often impractical in large-scale HBM studies due to field and logistical constraints. Nonetheless, the findings provide valuable reference data on recent exposure for this population. Notably, all urine samples contained at least one mycotoxin, indicating widespread exposure [50]. Furthermore, ten mycotoxins were detected in more than 10% of samples. Mean levels (P50 and/or AM) were used for comparison with existing international HBM data (see Supplementary Table S6).

As emerging compounds, mycotoxins have been detected in urine samples with varying frequencies, influenced by differences dietary habits, and the sensitivity of analytical methods. Additionally, both pre-harvest and post-harvest practices contribute to mycotoxin contamination along the food chain [84]. Pre-harvest factors include repeated cultivation, delayed planting, poor field sanitation, dense plating and inadequate waste management. Post-harvest contributors involve poor transport conditions, inadequate drying and shelling, suboptimal curing, storage on bare floors, and high ambient humidity. These factors likely account for the global variability observed in urinary mycotoxin levels.

Although HBM studies across Europe generally indicate that AFs are not the predominant mycotoxins to which populations are exposed [36,47], the present study found relatively high DFs for several AFs (DFs > 75%), with the exception of AFG_1_, which was detected in only 10.8% of samples. This contrasts with previous Spanish HBM studies [5,54], which reported DFs for AFs lower than 10%. The discrepancy is primarily attributed to the substantially lower LoQs used in the current study, underscoring that exposure to AFs, particularly AFB_1_, classified as “carcinogenic to humans” by IARC [83], remains relevant and should be minimized wherever possible.

Urinary AF levels, particularly AFM_1_, have also been detected in adult populations worldwide (see Supplementary Table S6). A higher proportion of participants in this study tested positive for AFB_1_. However, their concentrations were mostly lower than those documented in Chilean adults [22] and Spanish women [5], and similar to those observed in Portuguese adults [47] and Rwandan women [48]. These differences may be partly explained by the higher LoQs reported by Foerster et al. [22] and Dasí-Navarro et al. [5], which could have resulted in higher reported concentrations. In contrast, the LoQ reported by Martins et al. [47] and Collins et al. [48] were similar to that of the present study. AFM_1_, the primary metabolite of AFB_1_ [85], is commonly used as a biomarker in HBM studies to assess AFs exposure. Although high DFs for AFM_1_ have been reported elsewhere [19,44,48], our study documented the highest DF for this compound to date. Correspondingly, AFM_1_ concentrations exceeded those reported in most other global studies [19,29,37,44,45,47,48,49,50,86], except for Chilean adults [22], where the number of positive cases was limited. Other AFs such as AFB_2_, AFG_1_, and AFG_2_ have been reported in a few HBM studies in Spain [5,11], Portugal [47] and Rwanda [48], with concentrations comparable to or higher than those found in the present study.

ZEN and its primary metabolite (ZAN) were notably detected in urine samples, with ZAN present in over 57% of samples. Due to its estrogenic properties, ZEN has been extensively studied in HBM research, showing a broad range of DFs (6.9–100%). Prior to the current study, only one HBM investigation in Chinese adults [45] reported urinary concentrations of ZAN. While only a small proportion (7.7%) of those samples showed quantifiable levels, the concentrations were similar to those observed here. With respect to ZEN, comparable urinary levels have only been reported in the general population of Pakistan [19] and in Brazilian adults [37], these being among the lowest concentrations observed so far. In contrast, adults from Sweden [52], Germany [51] and Portugal [46], as well as the Italian general population [86] and Spanish participants from another HBM study [18], presented higher urinary ZEN levels than those found in this study.

OTA, classified by IARC as a potential human carcinogen, is another widely monitored mycotoxin in urine. Similarly To ZEN, HBM studies have reported OTA with highly variable DFs (1.2–100%) due to its potential bioaccumulation resulting from slow excretion. The present study found higher OTA levels than those reported in the general populations of Nigeria [44] and Brazil [37], as well as among women in Nigeria [29] and Rwanda [48]. Conversely, higher OTA concentrations have been reported in the general populations of Pakistan [19], women from Bangladesh [50], and adults from China [45,49], Sweden [52] and Belgium [36]. Urinary OTA levels in Portuguese adults [46] and the Italian general population [86] were comparable to those observed in the current study.

Regarding emerging mycotoxins, limited number of HBM studies reported urinary concentrations of them. AOH has only been detected in studies involving Chinese [31] and Portuguese [46] adults and the Nigerian general population [44], with levels substantially lower than those found here. Qiao et al. [31] also reported urinary concentrations of AME, which were similar to those observed in the present study. For CIT, higher levels were found in the Nigerian general population [44] and in pregnant women from Bangladesh [50] compared to BioMoVal participants, whose values were similar to those observed in Belgian [36] and Portuguese [46] adults. Notably, this is the first HBM study to report urinary concentrations of STER.

### 4.2. Statistical Relationships and Influence of Exposure Variables

In this study, we aimed to identify potential sources of mycotoxin exposure by examining the relationship between urinary concentrations of key mycotoxins (ΣAFs, ΣZENs, OTA and AOH) and relevant demographic, lifestyle and dietary variables. This analysis was particularly significant given the limited information available in the current scientific literature on such associations. Our statistical analysis revealed that certain demographic, lifestyle factors, and dietary habits, especially the recent consumption of meat and cereals, may influence mycotoxin exposure levels. Although modest, these results are consistent with this type of analysis given the complexity of influencing factors, and they provide valuable evidence to guide potential mitigation strategies and regulatory decisions. A more detailed discussion of these findings is presented below.

Sex was a relevant variable in our analysis, as statistically significant differences in urinary AFs levels were observed between male and female participants. However, these findings are not universally consistent with those of other HBM studies [22,31], which have not reported significant sex-based differences in mycotoxin exposure levels or prevalence. Socio-economic status is widely recognised as a key determinant in the accessibility and consumption of healthier food options [5]. In alignment with this, our regression analysis for AFs indicated that lower annual income was associated with increased exposure. Serasinghe et al. [87] suggested that parental education may exert a stronger influence on dietary patterns than the family income, but this variable is very difficult to account for.

Although younger individuals are often assumed to be at higher risk of mycotoxin exposure due to greater food intake [88], previous HBM studies have generally not found significant age-related differences in urinary mycotoxin levels [31,49]. In contrast, our findings demonstrated a statistically significant positive association between age and exposure to ΣZENs and OTA. This suggests that, beyond the consumption of food, other participant characteristics, like age, may play critical roles in mycotoxin exposure. Mycotoxins do not typically bioaccumulate due to their rapid excretion. However, while ZEN and its metabolites show very low tendency for accumulation in animal tissues [23], the slow elimination of OTA may lead to potential accumulation in the body [27], which could explain the correlation observed in the present study. Nevertheless, this interpretation remains challenging due to the limited available information.

Smoking status also emerged as a significant variable, with smokers exhibiting higher urinary levels of ΣAFs and OTA compared to passive and non-smokers. This may be attributable to generally poorer dietary habits among smokers, marked by higher processed food intake and lower consumption of healthy options [89]. Supporting this interpretation, a previous HBM study in Bangladesh found that the use of local stimulants, such as chewing tobacco, was associated with increased urinary OTA concentrations [50].

Furthermore, our data indicated that recent consumption of meat, particularly processed meat and chicken, and cereals was positively associated with urinary mycotoxin levels, particularly to AFs, ZENs and AOH. This observation is consistent with current understanding that the primary sources of dietary mycotoxin exposure include contaminated plant-based products and animal-based foods fed with contaminated feed. Specifically, cereal consumption is considered a major source of ZEN exposure [18,53], and significant correlations between cereal intake and urinary ZEN metabolites have been reported by Foerster et al. [22]. Additionally, although mycotoxin exposure usually stems from long-term consumptions; no other statistically significant associations between food consumption patterns and mycotoxin exposure were observed in our dataset. This may be due to the heterogeneous distribution of mycotoxins in food products, as well as the inherent limitations of self-reported dietary intake data obtained through food frequency questionnaires [90].

### 4.3. Risk Assessment

In the present study, PDI values were calculated for the entire study population in order to contextualise HBM results within a risk assessment framework, under the assumption that dietary intake constitutes the dominant exposure pathway to mycotoxins. Additionally, HQ and MOE values were determined to assess possible health risks associated with mycotoxin exposure.

The present findings revealed mean PDI values of AFs notably higher than those previously reported for adults in several countries, including Brazil (1 ng/kg-bw/day) [37], Chile (1.1 ng/kg-bw/day) [22], Portugal (13.4–16.7 ng/kg-bw/day) [47] and China (0.41 and 0.62 µg/kg-bw/day) [45,49], and for the general population of Italy (68 ng/kg-bw/day) [61] and Pakistan (43 ng/kg-bw/day) [19]. It is important to note that, with the exception of the study by Martins et al. [46], these investigations estimated PDI values exclusively based on urinary concentrations of AFB_1_ and/or AFM_1_, which may limit comparability. Within the Spanish context, only one HBM study, conducted by Pallarés et al. [11], reported PDI values for AFs that exceeded those observed in the current study. Across all referenced studies, the PDI values resulted in MOEs lower than the threshold of 10,000, indicating a potential public health concern.

With respect to ZEN exposure, while Brazilian adults [37] exhibited mean PDI values similar to those reported in our study, lower values were observed in the adult populations of China (0.039 µg/kg-bw/day) [45], Portugal (0.081 µg/kg-bw/day) [46] and Chile (6.00 µg/kg-bw/day) [22]. Based on the HQ approach, 16.5% of participants in the current study exceeded the established TDI, primarily due to elevated PDI values at P95, which resulted in HQs greater than 1. Comparatively, other HBM studies involving adults from China [49], Germany [51] and Italy [86] reported significantly lower proportions of participants exceeding the TDI for ZEN (0.88%, 1.67% and 0%, respectively). In contrast, higher percentages were found in Portugal [46] and Chile [22], where 24% and 100% of participants, respectively, surpassed the TDI, highlighting a greater level of concern. These discrepancies may be attributed to differences in dietary patterns, as well as the variability in toxicokinetics and toxicodynamic properties of the population [49].

Regarding OTA, similar to the pattern observed for ZEN, the mean PDI values for Brazilian adults [37] were comparable to those found in our study. However, the mean PDI values observed in this study were lower than those reported in Italy (0.139 µg/kg-bw/day) [61], China (0.22 and 0.14 µg/kg-bw/day) [45,49] and Bangladesh (0.400–0.426 µg/kg-bw/day) [50]. In contrast, Portuguese adults [46] demonstrated a slightly lower PDI value (0.005 µg/kg-bw/day) than that presented by our population, suggesting a comparable exposure among both countries. Although the Provisional Tolerable Weekly Intake (PTWI) of 0.12 µg/kg-bw/week reported by EFSA is no longer valid, a substantial percentage of participants in the current study (83.5%) exceeded this threshold. This finding aligns with previous observations by Xia et al. [19] and Solfrizzo et al. [86], who reported exceedance rates of 89% and 94% among Pakistani and Italian populations, respectively. Conversely, studies by Heyndrickx et al. [36] and Martins et al. [46] indicated significantly lower exceedance rates in Belgian (1%) and Portuguese (14%) adults, respectively. Notably, according to the most recent opinion issued by EFSA, the MOE approach is considered the most suitable method for the risk assessment of OTA [27]. Many of these studies also reported MOEs below 10,000, placing the exposure levels within a high-risk category, and underscoring significant health concerns related to OTA exposure.

The absence of established HBGVs and the insufficient toxicological data for any of the *Alternaria*-derived mycotoxins limits the reliability of risk assessment. At present, TTC values established by EFSA [32] are the only available approach for evaluating AOH. In this context, the mean exposure levels observed in our study exceeded the TTC threshold by a factor of 67 to 107. These findings align with the P95 PDI value for AOH in toddlers reported by EFSA, which was approximately 100-times higher than the established TTC value [32]. Such results are largely driven by the extremely low TTC value; therefore, their interpretation should be approached with caution, particularly as these values influence the HI calculations. Moreover, as no comparable data are currently available for the risk assessment of AOH in adult populations, direct comparisons with other studies were not possible. Finally, it is important to note that further toxicological data will be essential to establish HBGVs for emerging mycotoxins and enable more robust risk assessments.

Our findings are consistent with the growing body of evidence indicating that humans are routinely exposed to multiple mycotoxins simultaneously. Nevertheless, only a limited number of studies, such as that by Huang et al. [49], have conducted evaluations of the cumulative health risks arising from combined mycotoxin exposure, thus helping to avoid the underestimation of potential toxicological effects. Given that their specific combinations of mycotoxins do not align with those investigated in the present study, direct comparisons could not be made. Nonetheless, these findings clearly demonstrate that assessing individual mycotoxins in isolation is insufficient and likely underrepresents the actual health risks. Additionally, to ensure consistency in the risk assessment of chemical mixtures, it is essential that reference values for such mixtures are aligned with common toxicity endpoints, which may differ from those employed in the evaluation of individual compounds [91]. Finally, the cumulative assessment should be prioritized in future research and regulatory frameworks, warranting increased scientific attention and dedicated investigation.

## 5. Conclusions

This study provides an assessment of mycotoxin exposure levels in a representative sample of the Spanish adult population by quantifying urinary concentrations of twelve mycotoxins, encompassing both predominant and emerging compounds. Among the detected mycotoxins, AFs emerged as the most frequently identified compounds. However, HBM studies conducted previously across Europe have yielded inconsistent findings regarding their prevalence.

The present findings revealed that urinary mycotoxin levels in Spain were broadly comparable to those reported in other Southern European countries, particularly Portugal and Italy. Exposure levels were found to be significantly associated with several demographic and lifestyle factors, including sex, age, annual household income, and smoking status. Additionally, dietary intake within 24 h prior to sample collection, specifically the consumption of meat and cereals, was also linked to increased mycotoxin exposure.

To support health risk assessment, PDI values were estimated based on urinary biomarkers concentrations, and corresponding HQs and MOEs were calculated. Given the high frequency of co-exposure to multiple mycotoxins, HIs and MOETs were also computed to evaluate the potential risks posed by mixtures. The results suggest that the possibility of adverse health effects due to mycotoxin exposure cannot be excluded, highlighting the need for continued monitoring and risk assessment, especially due to the insufficient availability of HBGVs.

## Figures and Tables

**Table 1:** Socio-demographic characteristics, lifestyle and dietary habits of the studied population employed for building the regression models (N = 492).

Population’s Descriptors	N (%)	Median (Range)
Sex		
Male	261 (53.0)	−
Female	231 (47.0)	−
Age (years)	−	43 (18–65)
18–30	103 (21.0)	−
31–40	104 (21.1)	−
41–50	153 (31.1)	−
51–60	132 (26.8)	−
Home location (Province)		
Castellon	60 (12.2)	−
Valencia	261 (53.0)	−
Alicante	171 (34.8)	−
Annual per capita income (euros, €)		
<19,999	179 (36.4)	−
20,000–59,999	265 (53.8)	−
>60,000	48 (9.8)	−
Smoking status		
Non-smoker	60 (12.2)	−
Passive smoker	261 (53.0)	−
Smoker	171 (34.8)	−
Food consumed last 24 h (grams)		
Coffee	−	50 (0–400)
Nuts	−	0 (0–210)
Chocolate	−	0 (0–200)
Processed meat	−	0 (0–750)
Chicken	−	0 (0–500)
Fish	−	0 (0–2250)
Legumes	−	0 (0–375)
Rice	−	0 (0–375)
Cereals	−	0 (0–375)
Corn	−	0 (0–150)
Dairy products	−	155 (0–1340)

**Table 2:** Descriptive statistics of unadjusted and SG-adjusted urinary concentrations (ng·mL^−1^) of mycotoxins in the Spanish adult population (N = 492) *.

Biomarker	LoQ	DF (%)	P25	P50	AM	GM	P75	P95	Min.–Max.	SD
AFB_1_	0.005	76.0	0.005 (0.009)	0.04 (0.05)	0.08 (0.11)	0.02 (0.03)	0.08 (0.11)	0.21 (0.26)	<LoQ–8.16 (<LoQ–12.2)	0.38 (0.56)
AFB_2_	0.005	78.3	0.007 (0.012)	0.04 (0.05)	0.06 (0.07)	0.03 (0.03)	0.08 (0.10)	0.23 (0.25)	<LoQ–0.52 (<LoQ–0.61)	0.07 (0.09)
^a^ AFG_1_	0.005	10.8	−	−	0.06 (0.08)	−	−	−	<LoQ–0.34 (<LoQ–0.33)	−
AFG_2_	0.01	83.5	0.16 (0.19)	0.56 (0.69)	0.95 (1.25)	0.28 (0.36)	1.26 (1.66)	3.37 (4.45)	<LoQ–5.52 (<LoQ–20.8)	1.11 (1.70)
AFM_1_	0.01	89.0	0.05 (0.08)	0.30 (0.36)	0.47 (0.55)	0.18 (0.22)	0.71 (0.83)	1.48 (1.65)	<LoQ–4.84 (<LoQ–6.45)	0.55 (0.65)
^b^ ∑AFs	−	−	0.52 (0.64)	1.03 (1.26)	1.84 (1.99)	0.92 (1.17)	2.15 (2.71)	4.79 (6.04)	0.01–12.1 (0.03–21.4)	1.61 (2.27)
ZEN	0.01		<LoQ	<LoQ	0.01 (0.02)	<LoQ	<LoQ (0.01)	0.08 (0.11)	<LoQ–0.36 (<LoQ–0.35)	0.03 (0.04)
ZAN	0.01		<LoQ	0.04 (0.05)	0.57 (0.66)	0.03 (0.04)	0.50 (0.65)	2.05 (2.39)	<LoQ–46.2 (<LoQ–55.5)	2.53 (2.86)
^c^ ∑ZENs	−	−	<LoQ (0.01)	0.08 (0.11)	0.59 (0.68)	0.06 (0.08)	0.51 (0.66)	2.05 (2.40)	<LoQ–46.2 (<LoQ–55.5)	2.53 (2.86)
OTA	0.05		<LoQ	<LoQ	0.01 (0.06)	<LoQ	0.05 (0.07)	0.11 (0.16)	<LoQ–0.44 (<LoQ–0.64)	0.05 (0.06)
AOH	0.5		<LoQ	0.83 (1.11)	1.43 (1.70)	0.77 (0.98)	1.86 (2.08)	4.47 (4.74)	<LoQ–27.7 (<LoQ–35.0)	2.09 (2.45)
^a^ AME	0.5		−	−	0.84 (1.01)	−	−	−	<LoQ–1.87 (<LoQ–2.49)	−
^a^ CIT	0.5		−	−	0.56 (0.56)	−	−	−	<LoQ–0.68 (<LoQ–0.75)	−
^a^ STER	0.005		−	−	0.03 (0.04)	−	−	−	<LoQ–0.31 (<LoQ–0.34)	−

* SG-adjusted concentrations are presented into parenthesis. ^a^ Some parameters were not calculated due to the low DF (<20%). The presented parameters were calculated using only the positive samples (>LoQ). ^b^ ΣAFs = AFB_1_ + AFB_2_ + AFG_1_ + AFG_2_ + AFM_1_. ^c^ ΣZENs = ZEN + ZAN.

**Table 3:** Results of multiple linear regression model with influencing factors on the log-transformed concentrations of aflatoxins (∑AFs), sum of zearalenone biomarkers (∑ZENs), ochratoxin A (OTA) and alternariol (AOH) (N = 492).

Biomarker	Variable	Standardized Coefficients (β) (95% Confidence Interval)	*p*-Value *	R^2^
**ΣAFs**	Intercept	−22.174 (−29.083–−15.265)	**<0.001**	0.170
	Specific gravity	21.557 (14.784–28.330)	**<0.001**	
	Sex: Female	−0.160 (−0.246–−0.074)	**0.001**	
	Sex: Male	Ref.		
	Annual per capita income: <19,999 €	0.226 (0.073–0.379)	**0.004**	
	Annual per capita income: 20,000–59,999 €	0.156 (0.009–0.302)	**0.037**	
	Annual per capita income: >60,000 €	Ref.		
	Processed meat (kg in last 24 h)	0.657 (0.175–1.139)	**0.008**	
	Chicken (kg in last 24 h)	0.381 (0.036–0.727)	**0.031**	
	Smoking status: Smoker	0.109 (0.008–0.209)	**0.034**	
	Smoking status: Passive smoker	0.017 (−0.110–0.144)	0.796	
	Smoking status: Non-smoker	Ref.		
**ΣZENs**	Intercept	−14.842 (−30.127–0.442)	**0.047**	0.033
	Specific gravity	13.140 (−1.835–28.116)	**0.045**	
	Cereals (kg in last 24 h)	5.479 (0.949–10.009)	**0.018**	
	Age	0.009 (0.001–0.018)	**0.032**	
**OTA**	Intercept	−8.741 (−13.428–−4.054)	**<0.001**	0.068
	Specific gravity	6.907 (2.312–11.501)	**0.003**	
	Smoking status: Smoker	0.155 (0.088–0.223)	**<0.001**	
	Smoking status: Passive smoker	0.085 (−0.004–0.174)	0.063	
	Smoking status: Non-smoker	Ref.		
	Age	0.003 (0.001–0.006)	**0.014**	
**AOH**	Intercept	−26.213 (−33.037–−19.389)	**<0.001**	0.108
	Specific gravity	25.583 (18.893–32.273)	**<0.001**	
	Cereals (kg in last 24 h)	1.888 (−0.146–3.922)	**0.049**	

* *p*-values < 0.05 are highlighted in bold.

**Table 4:** Probable daily intakes (PDIs), reference values, hazard quotients (HQs), margins of exposure (MOEs), hazard indexes (HIs) and total margins of exposure (MOETs) for the studied population at GM and P95 distribution level.

		GM	P95
**ΣAFs**	PDI (µg/kg-bw/day)	1.27–2.03	6.26–9.77
	BMDL_10_ (µg/kg-bw/day	0.40	
	MOE	0.20–0.31	0.04–0.06
**ΣZENs**	PDI (µg/kg-bw/day)	0.01–0.02	0.36–0.65
	TDI (µg/kg-bw/day)	0.25	
	HQ	0.05–0.08	1.45–2.58
**OTA**	PDI (µg/kg-bw/day)	0.02–0.04	0.11–0.16
	BMDL_10_ (µg/kg-bw/day)	14.5	
	MOE	390–623	90–131
**AOH**	PDI (µg/kg-bw/day)	0.17–0.27	0.85–1.39
	TTC (µg/kg-bw/day)	0.0025	
	HQ	67–107	341–554
**Cumulative exposure**	**HI**	67–108	341–557
	**MOET**	0.20–0.31	0.04–0.06

## Data Availability

All data generated or analysed during this study are included in this published article.

## Supplementary Materials

The following supporting information can be downloaded at: https://www.mdpi.com/article/10.3390/toxics13100856/s1, Table S1: CAS numbers, transitions, adduct ions, collision energy, radiofrequency (RF) Lens and retention time (RT) of the target compounds and internal standards; Table S2: Descriptive statistics of Cre-adjusted urinary concentrations (µg·g Cre^−1^) of mycotoxins in the Spanish adult population (N = 492); Table S3: Spearman correlations between unadjusted urinary concentrations of mycotoxins; Table S4: Spearman correlations between SG-adjusted urinary concentrations of mycotoxins; Table S5: Spearman correlations between Cre-adjusted urinary concentrations of mycotoxins; Table S6: Urinary concentrations of mycotoxins in adult population worldwide; Figure S1: SRM chromatogram of each analysed compound in real urine samples.
