# Supplementary material for: Urinary Biomonitoring of Mycotoxins in Spanish Adults: Predictors of Exposure and Health Risk Evaluation
Source: Toxics. 2025 Oct 10;13(10):856. doi: 10.3390/toxics13100856 (PMC12567982; doi:10.3390/toxics13100856)
Supplement: Supplementary file 1 [file toxics-13-00856-s001.zip › toxics-3890812-supplementary.pdf]

toxics

MDPI

Article

Urinary Biomonitoring of Mycotoxins in Spanish Adults: Predictors of Exposure and Health Risk Evaluation

Borja Peris-Camarasa [1,2], Clara Coscollà [1,*], Pablo Dualde [1] and Olga Pardo [2,]

[1] Foundation for the Promotion of Health and Biomedical Research in the Valencian Region, FISABIO-Public Health, Avda. Cataluña, 21, 46020 Valencia, Spain; borja.peris@fisabio.es (B.P.-C.); pablo.dualde@fisabio.es (P.D.)
[2] Department of Analytical Chemistry, University of Valencia, Doctor Moliner, 50, 46100 Burjassot, Spain ; olga.pardo@uv.es (O.P.)
* Correspondence: clara.coscolla@fisabio.es ; Tel.: +34-961-926-333

Table of Contents

Type	Content	Page
Table S1	CAS numbers, transitions, adduct ions, collision energy, radiofrequency (RF) Lens and retention time (RT) of the target compounds and internal standards	2
Table S2	Descriptive statistics of Cre-adjusted urinary concentrations (µg·g Cre^{-1}) of mycotoxins in the Spanish adult population (N = 492).	3
Table S3	Spearman correlations between unadjusted urinary concentrations of mycotoxins.	3
Table S4	Spearman correlations between SG-adjusted urinary concentrations of mycotoxins.	4
Table S5	Spearman correlations between Cre-adjusted urinary concentrations of mycotoxins.	4
Table S6	Urinary concentrations of mycotoxins in adult population worldwide.	5
Figure S1	SRM chromatogram of each analysed compound in real urine samples.	9

Table S1. CAS numbers, transitions, adduct ions, collision energy, radiofrequency (RF) Lens and retention time (RT) of the target compounds and internal standards.

Compound	CAS Number	Internal Standard	Transitions (m/z) Precursor/Fragment	Adduct ion	Collision Energy (V)	RF Lens (V)	RT (min)
Aflatoxin M_1 (AFM_1)	6795-23-9	AFB_1-$^{13}C_{17}$	329.138 → 273.071[a]<br>329.138 → 229.030[b]	$[M+H]^+$	24.38 / 41.23	92	5.00
Aflatoxin G_2 (AFG_2)	7241-98-7	AFB_1-$^{13}C_{17}$	331.000 → 313.054[a]<br>331.000 → 245.130[b]	$[M+H]^+$	23.58 / 29.43	140	5.30
Aflatoxin G_1 (AFG_1)	1165-39-5	AFB_1-$^{13}C_{17}$	329.000 → 243.125[a]<br>329.000 → 311.071[b]	$[M+H]^+$	26.02 / 20.80	137	5.49
Aflatoxin B_2 (AFB_2)	7220-81-7	AFB_1-$^{13}C_{17}$	315.000 → 287.125[a]<br>315.000 → 259.125[b]	$[M+H]^+$	25.18 / 28.88	134	5.53
Aflatoxin B_1 (AFB_1)	1162-65-8	AFB_1-$^{13}C_{17}$	313.000 → 285.054[a]<br>313.000 → 269.071[b]	$[M+H]^+$	22.14 / 30.86	134	5.75
Alternariol (AOH)	641-38-3	OTA-$^{13}C_{20}$	259.150 → 185.042[a]<br>259.150 → 212.857[b]	$[M+H]^+$	30.78 / 28.76	128	5.90
Citrinin (CIT)	518-75-2	OTA-$^{13}C_{20}$	251.212 → 232.970[a]<br>251.212 → 205.071[b]	$[M+H]^+$	15.87 / 26.57	62	8.00
Zearalanone (ZAN)	5975-78-0	ZEN-$^{13}C_{18}$	319.100 → 275.137[a]<br>319.100 → 205.054[b]	$[M–H]^-$	21.22 / 23.79	110	8.55
Ochratoxin A (OTA)	303-47-9	OTA-$^{13}C_{20}$	404.000 → 239.042[a]<br>404.000 → 358.125[b]	$[M+H]^+$	23.11 / 13.38	120	8.55
Alternariol monomethyl eter (AME)	23452-05-3	OTA-$^{13}C_{20}$	273.150 → 199.000[a]<br>273.150 → 257.970[b]	$[M+H]^+$	30.19 / 27.66	142	8.60
Zearalenone (ZEN)	17924-92-4	ZEN-$^{13}C_{18}$	317.200 → 175.054[a]<br>317.200 → 273.208[b]	$[M–H]^-$	23.96 / 19.70	171	8.70
Sterigmatocystin (STER)	10048-13-2	OTA-$^{13}C_{20}$	325.000 → 281.042[a]<br>325.000 → 309.970[b]	$[M+H]^+$	37.77 / 24.97	88	9.65
**Internal Standards**							
Aflatoxin B_1-$^{13}C_{17}$ (AFB_1-$^{13}C_{17}$)	1217449-45-0	-	330.175 → 255.000[a]<br>330.175 → 301.018[b]	$[M+H]^+$	41.27 / 29.98	94	5.75
Ochratoxi A-$^{13}C_{20}$ (OTA-$^{13}C_{20}$)	911392-42-2	-	424.000 → 250.042[a]<br>424.000 → 377.125[b]	$[M+H]^+$	23.11 / 13.38	120	8.55
Zearalenone-$^{13}C_{18}$ (ZEN-$^{13}C_{18}$)	911392-43-3	-	335.200 → 185.054[a]<br>335.200 → 140.071[b]	$[M–H]^-$	23.96 / 29.52	171	8.70

[a]SRM transition used for quantification.

[b]SRM transition used for confirmation.

Table S2. Descriptive statistics of Cre-adjusted urinary concentrations (μg·g Cre^{-1}) of mycotoxins in the Spanish adult population (N = 492).

Biomarker	P25	P50	AM	GM	P75	P95	Min. – Max.	SD
AFB$_1$	0.007	0.03	0.08	0.02	0.07	0.20	<LoQ – 9.72	0.45
AFB$_2$	0.008	0.03	0.05	0.02	0.07	0.18	<LoQ – 0.62	0.07
[a]AFG$_1$	–	–	0.05	–	–	–	<LoQ – 0.23	–
AFG$_2$	0.13	0.49	0.87	0.25	1.23	3.05	<LoQ – 8.25	1.09
AFM$_1$	0.06	0.25	0.37	0.16	0.57	1.08	<LoQ – 2.82	0.39
[b]∑AFs	0.40	0.92	1.38	0.82	1.86	4.25	0.01 – 14.5	1.49
ZEN	<LoQ	<LoQ	0.01	<LoQ	<LoQ	0.07	<LoQ – 0.26	0.03
ZAN	<LoQ	0.04	0.43	0.03	0.45	1.58	<LoQ – 27.4	1.48
[c]∑ZENs	<LoQ	0.08	0.44	0.06	0.45	1.59	<LoQ – 27.4	1.48
OTA	<LoQ	<LoQ	<LoQ	<LoQ	0.05	0.13	<LoQ – 0.41	0.04
AOH	<LoQ	0.76	1.13	0.68	1.51	3.19	<LoQ – 15.2	1.33
[a]AME	–	–	0.68	–	–	–	<LoQ – 1.68	–
[a]CIT	–	–	<LoQ	–	–	–	<LoQ	–
[a]STER	–	–	0.03	–	–	–	<LoQ – 0.21	–

[a]Some parameters were not calculated due to the low DF (<20%). The presented parameters were calculated using only the positive samples (>LoQ).
[b]ΣAFs = AFB$_1$ + AFB$_2$ + AFG$_1$ + AFG$_2$ + AFM$_1$
[c]ΣZENs = ZEN + ZAN

Table S3. Spearman correlations between unadjusted urinary concentrations of mycotoxins.

	AFB$_2$	AFG$_1$	AFG$_2$	AFM$_1$	ZEN	ZAN	OTA	AOH	AME	CIT	STER
AFB$_1$	0.506**	0.215**	0.306**	0.265**	0.149**	-0.091*	0.148**	0.311**	0.053	0.151**	0.273**
AFB$_2$	–	0.192**	0.215**	0.429**	0.202**	0.013	0.196**	0.489**	0.062	0.135**	0.215**
AFG$_1$	–	–	-0.043	0.128**	0.121**	-0.074	0.229**	0.076	-0.039	0.244**	0.297**
AFG$_2$	–	–	–	0.412**	-0.008	0.058	0.068	0.170**	0.020	0.001	0.044
AFM$_1$	–	–	–	–	0.072	-0.003	0.213**	0.383**	0.062	0.097*	0.113*
ZEN	–	–	–	–	–	-0.023	0.124**	0.109*	-0.037	0.055	0.156**
ZAN	–	–	–	–	–	–	0.061	0.075	0.001	0.018	-0.184**
OTA	–	–	–	–	–	–	–	0.148**	-0.025	0.107*	0.208**
AOH	–	–	–	–	–	–	–	–	-0.013	-0.051	0.042
AME	–	–	–	–	–	–	–	–	–	-0.011	0.006
CIT	–	–	–	–	–	–	–	–	–	–	0.207**

*** Correlation is significant at the 0.01 level; * Correlation is significant at the 0.05 level.*

Table S4. Spearman correlations between SG-adjusted urinary concentrations of mycotoxins.

	AFB_2	AFG_1	AFG_2	AFM_1	ZEN	ZAN	OTA	AOH	AME	CIT	STER
AFB_1	0.476**	0.197**	0.321**	0.186**	0.155**	-0.109*	0.153**	0.263**	0.048	0.139**	0.291**
AFB_2	–	0.181**	0.197**	0.365**	0.192**	0.013	0.117**	0.427**	0.062	0.124**	0.237**
AFG_1	–	–	-0.061	0.109*	0.094*	-0.086	0.193**	0.056	-0.038	0.245**	0.297**
AFG_2	–	–	–	0.372**	0.010	0.027	0.074	0.148**	0.007	-0.019	0.057
AFM_1	–	–	–	–	0.021	-0.051	0.034	0.278**	0.058	0.088	0.120**
ZEN	–	–	–	–	–	-0.029	0.158**	0.096*	-0.037	0.041	0.164**
ZAN	–	–	–	–	–	–	0.054	0.037	0.001	0.006	-0.180**
OTA	–	–	–	–	–	–	–	0.070	-0.041	0.101*	0.227**
AOH	–	–	–	–	–	–	–	–	-0.023	-0.068	0.064
AME	–	–	–	–	–	–	–	–	–	-0.011	0.002
CIT	–	–	–	–	–	–	–	–	–	–	0.203**

*** Correlation is significant at the 0.01 level; * Correlation is significant at the 0.05 level.*

Table S5. Spearman correlations between Cre-adjusted urinary concentrations of mycotoxins.

	AFB_2	AFG_1	AFG_2	AFM_1	ZEN	ZAN	OTA	AOH	AME	CIT	STER
AFB_1	0.484**	0.178**	0.346**	0.173**	0.164**	-0.114*	0.185**	0.261**	0.046	0.133**	0.286**
AFB_2	–	0.163**	0.217**	0.334**	0.200**	-0.024	0.155**	0.410**	0.058	0.123*	0.222**
AFG_1	–	–	-0.078	0.105*	0.095*	-0.091*	0.185**	0.040	-0.039	0.243**	0.292**
AFG_2	–	–	–	0.365**	0.031	0.021	0.132**	0.154**	0.007	-0.022	0.060
AFM_1	–	–	–	–	0.010	-0.078	0.033	0.206**	0.062	0.083	0.124**
ZEN	–	–	–	–	–	-0.028	0.193**	0.095*	-0.037	0.041	0.155**
ZAN	–	–	–	–	–	–	0.057	0.020	-0.003	0.006	-0.190**
OTA	–	–	–	–	–	–	–	0.086	-0.040	0.100*	0.200**
AOH	–	–	–	–	–	–	–	–	-0.028	-0.072	0.047
AME	–	–	–	–	–	–	–	–	–	-0.011	0.003
CIT	–	–	–	–	–	–	–	–	–	–	0.202**

*** Correlation is significant at the 0.01 level; * Correlation is significant at the 0.05 level.*

Table S6. Urinary concentrations of mycotoxins in adult population worldwide.

Compound	Population	Age (years)	Sampling year	N	LoD ng·mL-1	LoQ ng·mL-1	DF (%)	AM, ng·mL-1 (µg·g Cre-1)	Median, ng·mL-1 (µg·g Cre-1)	P95, ng·mL-1 (µg·g Cre-1)	Range, ng·mL-1 (µg·g Cre-1)	Reference
AFB_1	Spanish adults	18 – 65	2021	492	0.002	0.005	76.0	0.11 (0.08)	0.05 (0.03)	0.29 (0.20)	<LoQ – 12.2 (<LoQ – 9.7)	This study
	Spanish mothers	36.5 ± 4	2007 – 2008	524	0.10	0.30	1.0	1.00	–	–	<LoQ – 1.20	Dasí-Navarro et al. 2023
	Chilean adults	38 – 74	2017	172	0.08	0.10	7.0	0.30	0.30	–	–	Foerster et al. 2021
	Rwandan women	18 – 55	–	138	–	0.002	8.0	0.01 (0.01)	0.01 (0.01)	–	<LoQ – 0.02 (<LoQ – 0.02)	Collins et al. 2021
	Portuguese adults	48.4 ± 15.2	2015 – 2016	94	0.02	0.05	11.7	<LoD	<LoD	0.023	–	Martins et al. 2020
AFB_2	Spanish adults	18 – 65	2021	492	0.002	0.005	78.3	0.07 (0.05)	0.05 (0.03)	0.25 (0.18)	<LoQ – 0.61 (<LoQ – 0.62)	This study
	Spanish mothers	36.5 ± 4	2007 – 2008	524	0.10	0.30	1.0	3.90	–	–	<LoQ – 9.90	Dasí-Navarro et al. 2023
	Spanish adults	–	2019 – 2020	56	1.50	5.00	32.0	5.30	–	–	<LoQ – 61.0	Pallarés et al. 2022
	Portuguese adults	48.4 ± 15.2	2015 – 2016	94	0.01	0.02	3.2	<LoD	<LoD	<LoD	–	Martins et al. 2020
AFG_1	Spanish adults*	18 – 65	2021	492	0.002	0.005	10.8	0.08 (0.05)	–	–	<LoQ – 0.33 (<LoQ – 0.23)	This study
	Spanish mothers	36.5 ± 4	2007 – 2008	524	0.10	0.30	1.0	3.00	–	–	<LoQ – 9.10	Dasí-Navarro et al. 2023
	Rwandan women	18 – 55	–	138	–	0.002	24.0	0.07 (0.04)	0.01 (0.01)	–	<LoQ – 1.22 (<LoQ – 0.42)	Collins et al. 2021
	Portuguese adults	48.4 ± 15.2	2015 – 2016	94	0.02	0.05	2.1	<LoD	<LoD	<LoD	–	Martins et al. 2020
AFG_2	Spanish adults	18 – 65	2021	492	0.003	0.010	83.5	1.25 (0.88)	0.69 (0.49)	4.45 (3.05)	<LoQ – 20.8 (<LoQ – 8.25)	This study
	Spanish adults	–	2019 – 2020	56	1.50	5.00	41.0	9.26	–	–	<LoQ – 69.4	Pallarés et al. 2022
	Portuguese adults	48.4 ± 15.2	2015 – 2016	94	0.01	0.02	3.1	<LoD	<LoD	<LoD	–	Martins et al. 2020

AFM_1	Spanish adults	18 – 65	2021	492	0.003	0.010	89.0	0.55 (0.37)	0.36 (0.25)	1.65 (1.08)	<LoQ – 6.45 (<LoQ – 2.82)	This study
	General population of Pakistan	4 – 80	2014	292	0.001	0.002	66.0	0.02	0.005	–	<LoD – 0.42	Xia et al. 2022
	Bangladesh pregnant women	–	2018 – 2019	447	0.10	0.30	0.22	0.42	–	–	–	Kyei et al. 2022
	Nigerian women	22 – 31	–	23	0.0003	0.001	11.0	0.10	0.08	–	<LoQ – 0.33	Braun et al. 2022
	Chilean adults	38 – 74	2017	172	0.80	1.10	1.0	1.80	1.80	–	–	Foerster et al. 2021
	Chinese adults	20 – 88	2019	227	–	–	2.20	0.35	–	–	<LoQ – 0.55	Huang et al. 2021
	Rwandan women	18 – 55	–	138	–	0.002	48.0	0.15 (0.10)	0.004 (0.004)	–	<LoQ – 1.40 (<LoQ – 0.66)	Collins et al. 2021
	Portuguese adults	48.4 ± 15.2	2015 – 2016	94	0.01	0.02	13.8	<LoD	<LoD	0.033	–	Martins et al. 2020
	Chinese adults	18 – 66	2017	260	0.03	0.10	10.4	0.25 (0.28)	–	–	<LoQ – 0.46 (<LoQ – 0.48)	Fan et al. 2019
	Brazilian adults	+18	2018	162	0.001	0.004	12.0	–	0.02	–	<LoD – 0.64	Franco et al. 2019
	Nigerian general population	–	2012	120	0.0003	0.001	72.5	0.04	0.01	–	<LoQ – 0.62	Šarkanj et al. 2018
	Italian general population	3 – 85	2011	52	–	0.020	6.0	0.07	0.10	–	<LoQ – 0.15	Solfrizzo et al. 2014
AOH	Spanish adults	18 – 65	2021	492	0.167	0.500	63.0	1.70 (1.13)	1.12 (0.76)	4.74 (3.19)	<LoQ – 35.0 (<LoQ – 15.2)	This study
	Chinese adults	17 – 75	2017	2212	–	0.06	10.4	0.193	–	–	<LoQ – 32.287	Qiao et al. 2022
	Portuguese adults	48.4 ± 15.2	2015 – 2016	94	0.40	0.90	13.0	–	<LoQ	2.40 (2.32)	–	Martins et al. 2019
	Nigerian general population	–	2012	120	0.010	0.030	6.7	0.06	0.03	–	<LoQ – 0.20	Šarkanj et al. 2018
AME	Spanish adults*	18 – 65	2021	492	0.167	0.500	1.2	1.01 (0.68)	–	–	<LoQ – 2.49 (<LoQ – 1.68)	This study
	Chinese adults	17 – 75	2017	2212	–	0.06	94.9	0.065 (0.050)	–	–	<LoQ – 2.022	Qiao et al. 2022

ZEN	Spanish adults	18 – 65	2021	492	0.003	0.010	23.2	0.02 (0.01)	<LoQ	0.11 (0.07)	<LoQ – 0.35 (<LoQ – 0.26)	This study
	General population of Pakistan	4 – 80	2014	292	0.010	0.020	37.0	16.7	<LoD	–	<LoD – 673	Xia et al. 2022
	Nigerian women	22 – 31	–	23	0.0003	0.001	83.0	0.26	0.18	–	<LoQ – 1.14	Braun et al. 2022
	Spanish adults	18 – 65	2019	40	0.33	1.00	40.0	6.70	–	–	<LoQ – 29.0	Carballo et al. 2021
	Chinese adults	20 – 88	2019	227	–	–	11.9	0.97 (0.65)	–	–	<LoQ – 18.4 (<LoQ – 10.9)	Huang et al. 2021
	Rwandan women	18 – 55	–	119	–	1.70	30.0	1.58 (1.42)	1.98 (1.01)	–	<LoQ – 3.77 (<LoQ – 4.78)	Collins et al. 2021
	Portuguese adults	48.4 ± 15.2	2015 – 2016	94	0.20	0.80	57.0	–	1.30 (1.01)	3.85 (5.38)	–	Martins et al. 2019
	Chinese adults	18 – 66	2017	260	0.05	0.10	6.9	0.15 (0.17)	–	–	<LoQ – 0.31 (<LoQ – 0.34)	Fan et al. 2019
	Brazilian adults	+18	2018	162	0.001	0.003	7.0	–	0.02	–	<LoD – 0.77	Franco et al. 2019
	Nigerian general population	–	2012	120	0.001	0.003	81.7	0.75	0.20	–	<LoQ – 20.0	Šarkanj et al. 2018
	German adults	16 – 65	–	60	0.010	0.025	100	0.10 (0.09)	0.07	–	<LoQ – 0.28	Ali and Degen 2018
	Swedish adults	–	2010 – 2011	252	0.002	0.007	36	0.12	0.10	–	<LoQ – 0.56	Mitropoulou et al. 2018
	Italian general population	3 – 85	2011	52	–	0.007	100	0.06	0.06	–	<LoQ – 0.12	Solfrizzo et al. 2014
ZAN	Spanish adults	18 – 65	2021	492	0.003	0.010	57.1	0.66 (0.43)	0.05 (0.04)	2.39 (1.58)	<LoQ – 55.5 (<LoQ – 27.4)	This study
	Chinese adults	18 – 66	2017	260	0.05	0.10	7.7	0.34 (0.43)	–	–	<LoQ – 1.82 (<LoQ – 2.24)	Fan et al., 2019
STER	Spanish adults	18 – 65	2021	492	0.002	0.005	16.3	0.04 (0.03)	–	–	<LoQ – 0.34 (<LoQ – 0.21)	This study

	Spanish adults	18 – 65	2021	492	0.017	0.050	29.5	0.06	<LoQ	0.16 (0.13)	<LoQ – 0.64 (<LoQ – 0.41)	This study
	General population of Pakistan	4 – 80	2014	292	0.001	0.002	99.0	0.14	0.08	–	<LoD – 4.82	Xia et al. 2022
	Bangladesh pregnant women	–	2018 – 2019	447	0.02	0.06	77.4	0.32	–	–	<LoQ – 2.93	Kyei et al. 2022
	Nigerian women	22 – 31	–	23	0.0003	0.001	56.0	0.01	0.01	–	<LoQ – 0.11	Braun et al. 2022
	Chinese adults	20 – 88	2019	227	–	–	3.52	0.35 (0.23)	–	–	<LoQ – 0.55 (<LoQ – 0.29)	Huang et al. 2021
	Rwandan women	18 – 55	–	138	–	0.02	71.0	0.02 (0.01)	0.03 (0.02)	–	<LoQ – 0.33 (<LoQ – 0.22)	Collins et al. 2021
OTA	Portuguese adults	48.4 ± 15.2	2015 – 2016	94	0.01	0.02	27.0	–	<LoQ	0.083 (0.062)	–	Martins et al. 2019
	Chinese adults	18 – 66	2017	260	0.05	0.10	1.20	0.36 (0.50)	–	–	<LoQ – 0.56 (<LoQ – 0.71)	Fan et al. 2019
	Brazilian adults	+18	2018	162	0.005	0.017	27.0	–	0.02	–	<LoD – 11.7	Franco et al. 2019
	Nigerian general population	–	2012	120	0.0003	0.001	78.3	0.05	0.04	–	<LoQ – 0.31	Šarkanj et al. 2018
	Swedish adults	–	2010 – 2011	252	0.002	0.006	51.0	1.20	0.87	–	<LoQ – 16.5	Mitropoulou et al. 2018
	Belgian adults	19 – 65	2013 – 2014	239	0.001	–	35.0	27.8 (36.4)	15.2 (15.5)	–	<LoQ – 368 (<LoQ – 927)	Heyndrick et al. 2015
	Italian general population	3 – 85	2011	52	–	0.006	100	0.14	0.06	–	<LoQ – 2.13	Solfrizzo et al. 2014

CIT	Spanish adults*	18 – 65	2021	492	0.167	0.50	1.0	0.56	–	–	<LoQ – 0.75	This study
	Bangladesh pregnant women	–	2018 – 2019	447	0.17	0.50	29.3	2.46	–	–	<LoQ – 14.5	Kyei et al. 2022
	Portuguese adults	48.4 ± 15.2	2015 – 2016	94	0.50	1.00	13.0	–	<LoQ	–	–	Martins et al. 2019
	Nigerian general population	–	2012	120	0.003	0.01	65.0	5.96	0.84	–	<LoQ – 241	Šarkanj et al. 2018
	Belgian adults	19 – 65	2013 – 2014	239	0.001	–	59.0	0.057 (0.074)	0.018 (0.020)	–	<LoQ – 1.40 (<LoQ – 1.50)	Heyndrick et al. 2015

*The presented descriptive statistics were calculated using only the positive samples (<LoQ).

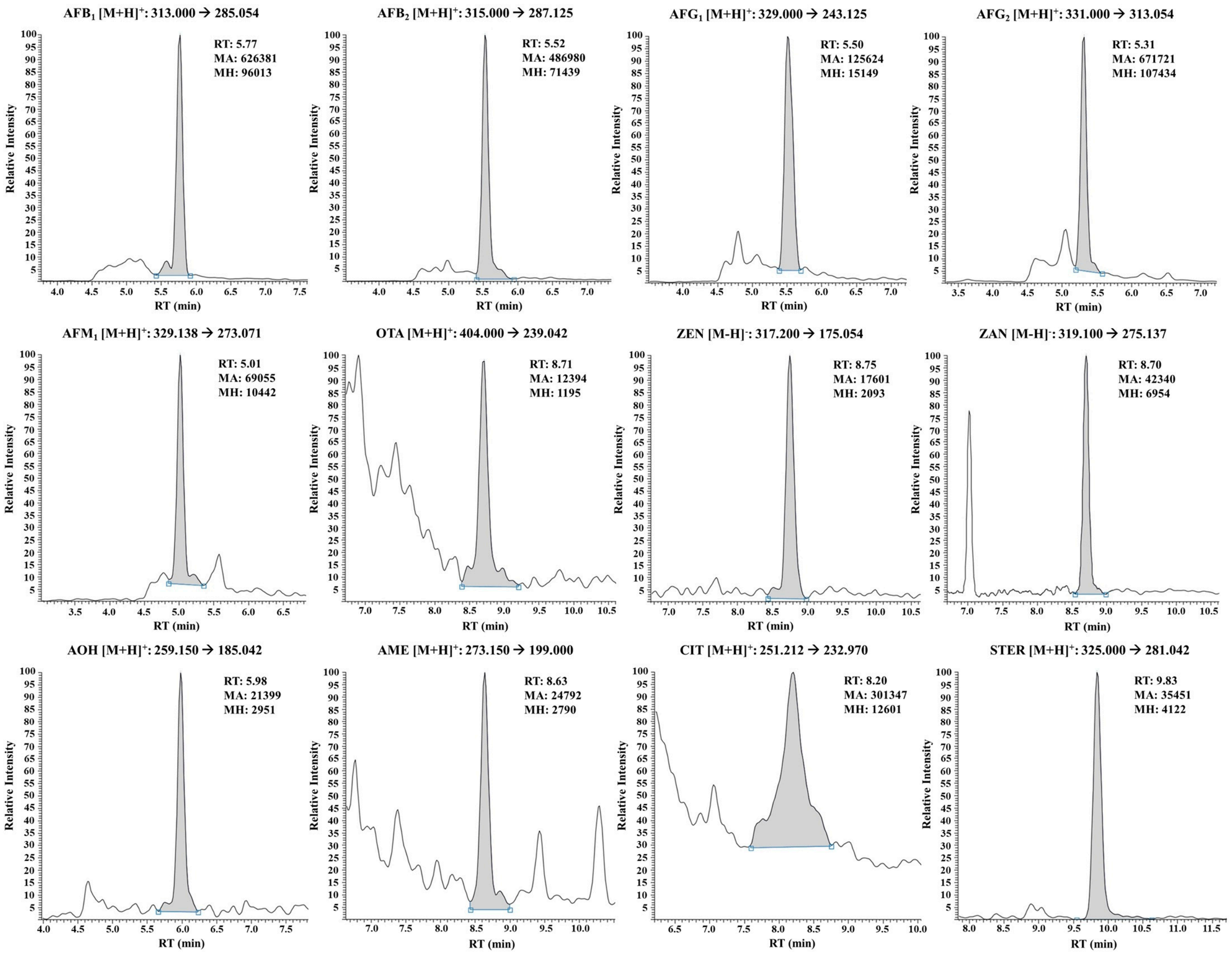

Figure S1. SRM chromatogram of each analysed compound in real urine samples.
